# Effect of Various Invitation Schemes on the Use of Fecal Immunochemical Tests for Colorectal Cancer Screening: Protocol for a Randomized Controlled Trial

**DOI:** 10.2196/16413

**Published:** 2020-04-03

**Authors:** Laura Fiona Gruner, Michael Hoffmeister, Leopold Ludwig, Hermann Brenner

**Affiliations:** 1 Division of Clinical Epidemiology and Aging Research German Cancer Research Center Heidelberg Germany; 2 Medical Faculty Heidelberg University of Heidelberg Heidelberg Germany; 3 Gastroentereologische Schwerpunktpraxis Dornstadt Germany; 4 Division of Preventive Oncology German Cancer Research Center and National Center for Tumor Diseases Heidelberg Germany; 5 German Cancer Consortium, German Cancer Research Center Heidelberg Germany

**Keywords:** colorectal cancer, early detection, screening, fecal immunochemical test (FIT), invitation, Germany

## Abstract

**Background:**

Fecal occult blood testing has been offered for many years in the German health care system, but participation rates have been notoriously low.

**Objective:**

The aim of this study is to evaluate the effect of various personal invitation schemes on the use of fecal immunochemical tests (FITs) in persons aged 50-54 years.

**Methods:**

This study consists of a three-armed randomized controlled trial: (1) arm A: an invitation letter from a health insurance plan including a FIT test kit, (2) arm B: an invitation letter from a health insurance plan including an offer to receive a free FIT test kit by mail upon easy-to-handle request (ie, by internet, fax, or reply mail), and (3) arm C: an information letter on an existing colonoscopy offer (ie, control). Within arms A and B, a random selection of 50% of the study population will receive reminder letters, the effects of which are to be evaluated in a substudy.

**Results:**

A total of 17,532 persons aged 50-54 years in a statutory health insurance plan in the southwest of Germany—AOK Baden-Wuerttemberg—were sent an initial invitation, and 5825 reminder letters were sent out. The primary end point is FIT usage within 1 year from receipt of invitation or information letter. The main secondary end points include gender-specific FIT usage within 1 year, rates of positive test results, rates of colonoscopies following a positive test result, and detection rates of advanced neoplasms. The study was launched in September 2017. Data collection and workup were completed in fall 2019.

**Conclusions:**

This randomized controlled trial will provide important empirical evidence for enhancing colorectal cancer screening offers in the German health care system.

**Trial Registration:**

German Clinical Trials Register (DRKS) DRKS00011858; https://bit.ly/2UBTIdt

**International Registered Report Identifier (IRRID):**

DERR1-10.2196/16413

## Introduction

### Background

Annual or biennial screening by fecal occult blood tests (FOBTs) has been shown by randomized clinical trials to reduce colorectal cancer (CRC) mortality by up to 30% [1]. These effects were achieved even with guaiac-based FOBTs (gFOBTs), which had limited sensitivity in detecting CRC and its precursors. Even stronger effects are to be expected by screening with immunochemical FOBTs (iFOBTs), often called fecal immunochemical tests (FITs), which have substantially higher sensitivity than gFOBTs [2]. FITs are now commonly recommended for CRC screening by national and international guidelines [2-4], and are increasingly offered for CRC screening in many countries [5,6]. However, participation rates in screening have remained low in countries where screening is offered in an opportunistic manner without targeted invitation of the eligible population [5]. This particularly applies to Germany, where gFOBT screening had been offered from 1977 to March 2017; FIT-based screening has been offered from age 50 years on since April 2017. For conducting FITs that are covered by the health insurance system, people have to pick up and return the tests at medical practices. Although personal information letters on CRC screening offers have been sent to the eligible population since July 2019 [3], they do not include FITs or specific low-threshold access to FITs, which are deemed to be crucial to achieve high participation rates [7,8]. The aim of this trial is to assess the effect of various invitation schemes on use of FITs for CRC screening in routine practice in the German health care system.

### Objectives

The primary outcome that will be investigated is as follows: determine the proportion of people completing a FIT within 1 year after receiving a personal invitation letter within each trial arm.

Secondary outcomes are to determine the following:

1. The rate of positive test results.
2. The rate of performing a colonoscopy after a positive test.
3. The rate of performing a colonoscopy after a negative test.
4. The rate of discovered advanced colorectal neoplasia (ie, advanced adenomas and cancer).
5. The positive predictive value of the test.
6. The rate of performing a screening colonoscopy, in general, within 1 year.

Gender-specific analyses will be conducted.

## Methods

### Setting and Design

This protocol follows the Standard Protocol Items: Recommendations for Interventional Trials (SPIRIT) 2013 Statement for clinical trial protocols [9] and the Fecal Immunochemical TesTs for Hemoglobin Evaluation Reporting (FITTER) guidelines [10].

The study is being conducted by the German Cancer Research Center (DKFZ) in cooperation with a statutory health insurance plan in the southwest of Germany: AOK Baden-Wuerttemberg (AOK BW).

The study is designed as a three-armed randomized controlled trial, in which 17,532 people aged 50-54 years are randomly selected to receive a letter from their health insurance plan (ie, AOK BW) with the following: (1) arm A: an invitation letter including a FIT test kit, (2) arm B: an invitation letter including an offer to receive a free FIT test kit by mail upon easy-to-handle request (ie, via Internet, fax, or reply mail), or (3) arm C: an information letter on CRC screening in the form of a colonoscopy; the control group represents routine practice with no study-related adaption of the letter. There are about 5844 insurants per arm. In addition, a reminder letter is being sent by random selection to 50% of the population in arms A and B. Prior to the recruitment of participants, the study, which was launched in 2017, was approved by the ethics committee of the Medical Faculty of the University of Heidelberg, Germany. It was registered in the German Clinical Trials Register (DRKS) on March 20, 2017 (DRKS-ID: DRKS00011858) with the following title: *Increase of the usage and effectiveness of colorectal cancer screening by means of targeted invitations with and without providing fecal immunochemical tests*. Written informed consent is being obtained from all participants.

Regardless of the FITs offered to participants in arms A and B within the intervention groups, the entire targeted population has access to CRC screening offers in routine practice according to law. However, only the opportunistic screening program is available at the time of study recruitment: CRC screening comprises annual testing for blood in the stool using the FIT or by performing a screening colonoscopy from age 50 years for men and women within a specific health insurance plan of AOK BW called *AOK-FacharztProgramm* (AOK Specialist Program); screening colonoscopy is otherwise offered from age 55 years in Germany during the recruitment period.

**Figure 1:**
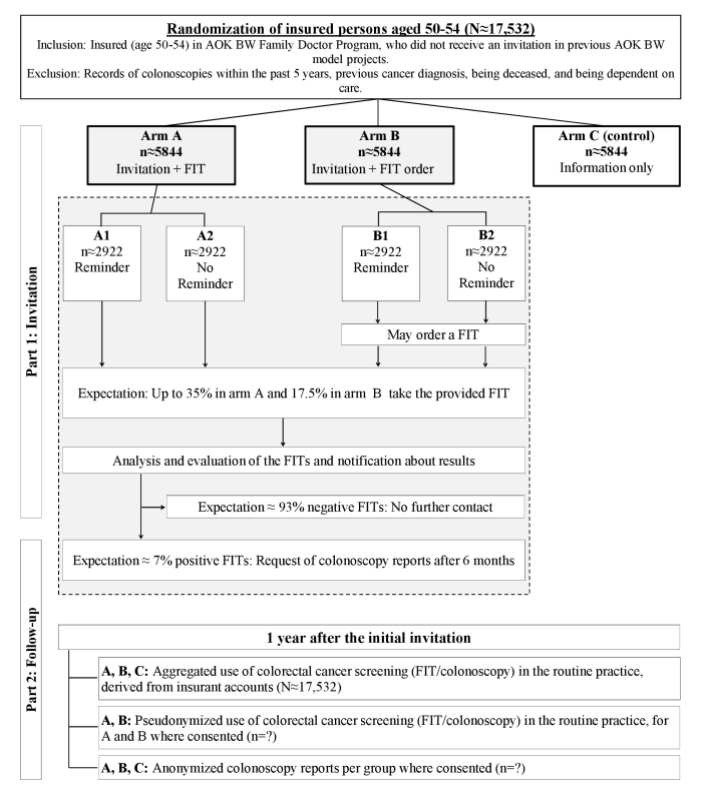
Study design. Total expected fecal immunochemical test (FIT) use per study arm: A=35%, B=17.5%, and C=10%. Participants in arms A and B may receive a FIT as part of the intervention or can additionally request it in routine practice. Participants in arm C (ie, control) can only use the FIT in routine practice. Data on the use of screening methods until 1 year after the initial invitation will be collected. AOK BW: AOK Baden-Wuerttemberg; A1 and A2: subgroups of arm A; and B1 and B2: subgroups of arm B.

The study consists of two parts: (1) the mailing of different personal invitation letters for CRC screening, with low-threshold access to a FIT in the intervention arms, and (2) the follow-up of the use and outcome of a colonoscopy after a positive FIT, as well as the assessment of conducted FITs and colonoscopies in routine practice within 1 year after the initial invitation. Routine FIT and colonoscopy usage is derived from AOK BW claims data, which is being aggregated and pseudonymized where consent was obtained. All collected information is being stored and monitored in a study database by trained staff. The study design and the study assessments are shown in Figure 1 and Table 1, respectively.

**Table 1:** Overview of study assessments at relevant time points.

Assessments		T0 (0 wk)	T1								T2 (6 months after positive FIT^a^)	T3 (1 year)
			2 wk	4 wk	6 wk	8 wk	10 wk	12 wk	14 wk	>14^b^ wk		
**Randomization and initial invitations**												
	Randomization^c^	✓	–^d^	–	–	–	–	–	–	–	–	–
	Subrandomization^e^	✓	–	–	–	–	–	–	–	–	–	–
	Tranche 1	✓	–	–	–	–	–	–	–	–	–	–
	Reminder 1	–	–	✓	–	–	–	–	–	–	–	–
	Tranche 2	–	✓	–	–	–	–	–	–	–	–	–
	Reminder 2	–	–	–	✓	–	–	–	–	–	–	–
	Tranche 3	–	–	✓	–	–	–	–	–	–	–	–
	Reminder 3	–	–	–	–	✓	–	–	–	–	–	–
	Tranche 4	–	–	–	✓	–	–	–	–	–	–	–
	Reminder 4	–	–	–	–	–	✓	–	–	–	–	–
	Tranche 5	–	–	–	–	✓	–	–	–	–	–	–
	Reminder 5	–	–	–	–	–	–	✓	–	–	–	–
	Tranche 6	–	–	–	–	–	✓	–	–	–	–	–
	Reminder 6	–	–	–	–	–	–	–	✓	–	–	–
**Laboratory analysis of the provided FITs and notification about results**												
	Laboratory analysis	–	✓	✓	✓	✓	✓	✓	✓	✓	–	–
	Notification	–	✓	✓	✓	✓	✓	✓	✓	✓	–	–
Follow-up of colonoscopy use after a positive FIT: request reports		–	–	–	–	–	–	–	–	–	✓	–
**Use of colorectal cancer screening in routine practice within 1 year**												
	Use of FITs^f^	–	–	–	–	–	–	–	–	–	–	✓
	Use of colonoscopy^f^	–	–	–	–	–	–	–	–	–	–	✓
	Colonoscopy reports^g^	–	–	–	–	–	–	–	–	–	–	✓

^a^FIT: fecal immunochemical test.

^b^Duration depends on how long insurants are sending back the FITs.

^c^Arm A: invitation + FIT; arm B: invitation + order; and arm C: information only (ie, control).

^d^Not applicable.

^e^Subrandomization in arms A and B: 50% receive reminder.

^f^Aggregated for all arms and additionally pseudonymized for arms A and B where consent was given.

^g^Anonymized for all arms where consent was given.

### Inclusion and Exclusion Criteria

Inclusion criteria for receiving a letter are as follows: (1) aged 50-54 years, (2) enrolled in the AOK Family Doctor Program (HausarztProgramm) of AOK BW (Hausarztzentrierte Versorgung [HZV] contract: § 73b SGB V), and (3) did not receive an invitation in previous rounds of AOK BW model projects in previous years. Exclusion criteria for receiving a letter are as follows: (1) had an insurance-recorded colonoscopy within the past 5 years, (2) had a previous cancer diagnosis, (3) being deceased, and (4) being dependent on care.

Participants with a positive FIT result detected with the provided test kit are contacted by the DKFZ during the second part of the study to follow up on usage and outcome of colonoscopies.

### Part 1: Personal Invitation for Colorectal Cancer Screening

The intervention is a personal invitation letter to perform a commercially available and validated FIT for quantitative detection of human hemoglobin (Hb); the test used is the OC-Sensor FIT (Eiken Chemical, Tokyo, Japan). Study information is being embedded in a mandatory, insurance-related information letter about the offer to undergo colonoscopies from age 50 years, within the AOK Specialist Program. Suitable insured persons are being randomized into three arms—A, B, and C (ie, control)—by AOK BW (randomization was performed using a random number generator: structured query language [SQL] statement, database management system [DBMS]_RANDOM). Letters are being sent out biweekly in six tranches—one-sixth of each arm per tranche. A second randomization is dividing arms A and B into two respective subgroups—arm A: A1 and A2; arm B: B1 and B2. Subgroups A1 and B1 get a reminder letter after 4 weeks. No additional FIT kit is attached to the reminder letter and it states that the reminder is invalid if the FIT offer was already taken or ordered.

Within intervention arm A, insured persons receive a personal invitation letter, which describes CRC screening methods and includes a free FIT in a prepacked test kit. This kit contains one FIT, written and graphical instructions, two stool sample collectors, a record sheet for date of sample taking and year of birth, and a return envelope for mailing the test to the study center at DKFZ. Furthermore, the study information sheet, with optional email or phone consultation by the DKFZ, and the consent form are included.

Within intervention arm B, insured persons are also sent a personal invitation letter describing CRC screening methods, with the additional offer to order a FIT for free from the DKFZ via reply mail, fax, email, or online form. The study information sheet is added, but the consent form is only sent with the prepacked test kit upon FIT request.

Within control arm C, insured persons receive the insurance-related information letter mentioning a colonoscopy but do not receive the FIT itself nor the offer to order a FIT from the DKFZ. They may, however, make use of the FIT in routine practice: aggregated data on routine use is being provided by AOK BW.

Insurants randomized to arms A and B, who decide to take the FIT offer, are asked to sign the informed consent form. By signing this, they agree to the FIT analysis; to receive a written test result, which will also be sent to the family physician if not contradicted; to data storage and evaluation (ie, FIT result, age, gender, and first three digits of postal code); and to be recontacted during follow-up in case of a positive result. Optionally, they can allow the DKFZ to receive pseudonymized claims data from CRC screening participation in routine practice within 1 year after the initial invitation.

As proposed by the FIT manufacturer, participants are being instructed to spread the tip of the test stick over the freshly passed whole feces at various points until the tip’s grooves were filled with feces, and then to reinsert the probe into the device. The serrated probe that is attached to the device cap collects 10 mg feces into 2 mL of buffer. The FIT and the informed consent sheet are mailed to the DKFZ in a postpaid envelope. Upon arrival at the DKFZ, the FITs are stored in a fridge and separated from person-identifying information (ie, pseudonymized). The FITs are then sent by cooled transport to an external certified laboratory: Labor Limbach, Heidelberg, Germany, DIN EN ISO 15189 accredited. Trained laboratory personnel blinded to the randomization arm perform the analysis in a fully automated manner using the original OC-Sensor Pledia FIT device. Samples are disposed of after the analysis. As recommended by the manufacturer, the cutoff for a test result to be positive is 50 ng Hb/mL buffer (equal to 10 µg Hb/g feces). Results above the upper analytical limit of 1000 ng Hb/mL buffer are not diluted and not retested. Collection, arrival, and analysis dates of fecal samples are recorded. The DKFZ receives the laboratory reports, each checked and signed by a certified medical doctor. Additionally, the quantitative test results of the FITs are transferred electronically to the DKFZ, using DocNet plus, version 4 (DocNet Systems GmbH), where they are integrated into the study database.

Missing informed consent forms are being requested before any test analysis starts. Participants with failed tests receive a new test kit (eg, due to failed sampling or if the difference between the day of sample taking and arrival at the DKFZ is more than 7 days without cooling, followed by a negative test result).

The DKFZ sends a notification about the qualitative test result (ie, positive or negative, based on the manufacturer's recommended threshold) to the participants in an understandable manner. With a positive result, a consultation with the general practitioner is recommended to consider a colonoscopy for further examination. A copy of the laboratory report is sent to the family physician, unless participants did not wish so.

### Part 2: Follow-Up of the Use of Screening Methods

A total of 6 months after a positive test result, the DKFZ contacts the respective participants in arms A and B to request permission for obtaining the reports of any subsequently conducted colonoscopies. Separate information sheets and consent forms are being used and the name of the treating gastroenterologist is requested. Relevant information from colonoscopy reports are being extracted and entered into an electronic database by two independent, trained data extractors and checked for inconsistencies.

Claims data of screening colonoscopies and FITs conducted within 1 year after the initial invitation or information letter is being provided by AOK BW in an aggregated manner per study arm (ie, A, B, and C) and gender. Individual pseudonymized claims data regarding the usage of FITs and colonoscopies is available for participants in the intervention arms upon specific informed consent.

In addition, results of screening colonoscopies in the age group of 50-54 years is being documented by the gastroenterologists in standardized survey forms, which are mandatory for billing. The billing company *MEDIVERBUND AG* captures the data electronically, anonymizes it, and sends it to the DKFZ for the analysis. Assignment to the initial study arms—A, B, or C—is possible only after consent.

### Sample Size Calculation

The study population can be drawn in a very efficient manner from AOK BW. Approximately 17,532 insured persons met the inclusion criteria and received an initial letter: 5844 persons per arm—A, B, and C. A reminder is being sent out to 50% of the people in arms A and B—2922 persons per subgroups A1 and B1—after 4 weeks.

The following expectations are based on experiences from a previous model project [8], taking into account changes in this study’s conditions. The expected usage of a FIT for both genders combined is about 10% for the people in the control group (ie, FIT use in routine practice). In intervention arm A (ie, invitation with FIT), a 3.5-fold increase to 35% (ie, about 30% after the first letter plus about 5% after the reminder) is expected. In intervention arm B (ie, invitation with offer to order a FIT), a 1.75-fold increase to about 17.5% is expected (ie, about 15% after the first letter plus about 2.5% after the reminder). The sample size—approximately 5844 per arm—allows a high-precision estimation of the expected relative increase in FIT usage; the expected 95% CIs for relative FIT usage are 3.22-3.81 and 1.59-1.92 in intervention arms A and B, respectively, compared to the control arm. The statistical power to detect an effect within the expected range is close to 100% for both types of intervention: SAS, version 9.4 (SAS Institute) POWER procedure, two-sided Pearson chi-square test with significance level alpha=.017. For the comparison of the three arms—A, B, and C—we adjusted for multiple testing according to the Bonferroni-Holm method. For the most stringent adjustment with alpha=.017 (ie, baseline alpha=.05 with three tests), there is still excellent power.

In the randomized substudy, the increase in FIT usage due to a reminder letter in comparison to the one-time invitation will be analyzed. The expected FIT usage in arm A is approximately 40% with the reminder compared to 30% without the reminder; the expected FIT usage in arm B is approximately 20% with the reminder compared to 15% without the reminder. The included sample size of the randomized substudy (ie, 2922 per arm) allows for a precise estimation of the impact of sending a reminder. The expected relative FIT use (95% CI) in subgroups A1 and B1 (ie, received a reminder) compared to the respective subcontrol groups A2 and B2 (ie, one-time invitation only) would be 1.33 (1.24-1.43) and 1.33 (1.19-1.49), respectively. The statistical power to detect an effect of the expected order of magnitude is close to 100%: SAS, version 9.4 (SAS Institute) POWER procedure, two-sided Pearson chi-square test with significance level alpha=.025. For the two comparisons (ie, A1 vs A2 and B1 vs B2), multiple testing was adjusted for, according to the Bonferroni-Holm method. For the most stringent adjustment with alpha=.025 (ie, baseline alpha=.05 with two tests) there is still excellent power.

### Statistical Analysis

The statistical analysis of this confirmatory study primarily involves comparing the use of FITs in the intervention arms and the control arm within 1 year after the initial letter. In addition, within the intervention arms, the effect of a one-time invitation will be compared to an invitation with a reminder letter.

Comparisons will be done by two-sided chi-square tests for differences in the participation rates. Secondary outcomes will be addressed by descriptive and exploratory analyses. Data for the primary outcome analysis are expected to be complete, as complete information on FITs conducted in routine practice is obtained through insurance claims data (ie, billing codes for laboratory analysis of the FIT), and complete information on FITs conducted through the special offers is directly available from the study center.

## Results

In total, 17,532 invitation letters and 5825 reminder letters were sent out; the study was launched in September 2017. Data collection and workup were completed in fall 2019.

## Discussion

We initiated this three-armed randomized controlled trial in order to evaluate, with the highest possible evidence [11,12], the effect of low-threshold invitation schemes on the usage of CRC screening—with focus on FITs—in the 50-54-year-old population.

CRC remains one of the most frequent causes of cancer and reasons for cancer-related death in Germany [13] and worldwide [14]. Participation rates of CRC screening need to be increased; various approaches have recently been investigated in different countries in a rising number of population-based [7] and randomized trials [8,15-17]. Among those are results of a nationwide, FIT-based screening program in the Netherlands [7], as well as from a previously conducted model project in Germany [8]; these studies have consistently shown that the usage of a stool test increases after the target population receives personal invitations including the test. Despite that, the nationwide organized invitation procedure, which was introduced in Germany in 2019 to improve CRC screening, only comprises a personal invitation sent out by health insurance plans, with an enclosed gender-specific information brochure but no direct provision of, or low-threshold access to, a FIT [3]. Moreover, first trends indicate that the use of a stool test has further declined since the change from gFOBT to the more sensitive and widely recommended FITs in 2017 [18-20].

Usage of the available screening methods for CRC reduces the incidence and the mortality of CRC by removal of precursors and detection of cancer at an early stage [1,21] and, thereby, reduces the costs associated with CRC-related therapies. Furthermore, colonoscopies following a positive FIT result might be more effective compared to colonoscopies without a previous FIT, due to a higher chance of detecting relevant findings, such as advanced adenomas. Thus, an invitation procedure with low-threshold FIT provision could not only increase the usage of the test, but also improve the effectiveness of subsequent colonoscopies, thereby avoiding potential adverse outcomes and costs arising in the case of later detection of CRC.

The concept of this study allows for monitoring and investigating the usage rate of FITs following a once-only CRC screening information letter in routine practice and after invitation with provision of the FIT; whether a colonoscopy was conducted after the FIT, no matter if there was a positive or negative FIT outcome; as well as the outcome of such a colonoscopy. This might lead to a better understanding of the screening-related and possibly gender-specific actions taken as well as the needs in the targeted population. Findings from colonoscopy reports might further help to point out the relevance for screening, due to the prevalence of advanced adenomas in this age group.

All invited persons are between 50 and 54 years of age, representing the age group for whom the FIT is covered in the national screening program. Nevertheless, selection bias due to the focus of our study on AOK-insured persons enrolled in the AOK Family Doctor Program cannot be ruled out, which may limit external validity of the study.

To our knowledge, this is the first study that examines a low-threshold order option of the FIT, in addition to direct provision of a FIT, included in the invitation letter. The concept of personal invitations being sent out by a health insurance plan is currently implemented in Germany [3]; however, it includes neither direct provision of a FIT with the invitation letter nor a low-threshold order option. The results of this randomized controlled trial will, therefore, provide important empirical evidence for potential further enhancement of CRC screening in routine practice in the German health care system and beyond.

## Abbreviations

AOK BW: AOK Baden-Wuerttemberg
CRC: colorectal cancer
DBMS: database management system
DKFZ: German Cancer Research Center
DRKS: German Clinical Trials Register
FIT: fecal immunochemical test
FITTER: Fecal Immunochemical TesTs for Hemoglobin Evaluation Reporting
FOBT: fecal occult blood test
gFOBT: guaiac-based fecal occult blood test
Hb: hemoglobin
HZV: Hausarztzentrierte Versorgung
iFOBT: immunochemical fecal occult blood test
SPIRIT: Standard Protocol Items: Recommendations for Interventional Trials
SQL: structured query language
